# Mulberry Leaf Polyphenols and Fiber Induce Synergistic Antiobesity and Display a Modulation Effect on Gut Microbiota and Metabolites

**DOI:** 10.3390/nu11051017

**Published:** 2019-05-06

**Authors:** Qian Li, Fan Liu, Jun Liu, Sentai Liao, Yuxiao Zou

**Affiliations:** 1 Guangdong Key Laboratory of Agricultural Products Processing, Sericultural & Agri-Food Research Institute, Guangdong Academy of Agricultural Sciences, Guangzhou 510610, China; liqian.hubei@163.com (Q.L.); liufan@gdaas.cn (F.L.); liuj@gdaas.cn (J.L.); liaost@163.com (S.L.); 2 Key Laboratory of Functional Food, Ministry of Agriculture and Rural Affairs, Sericultural & Agri-Food Research Institute, Guangdong Academy of Agricultural Sciences, Guangzhou 510610, China

**Keywords:** mulberry leaf, obesity, gut microbiota, metabolomics

## Abstract

The antiobesity molecular mechanisms of mulberry leave components were analyzed based on intestinal micro-ecology and metabolomics. An obesity model was established by feeding rats with a high-calorie diet. Rats were divided into seven groups: the obesity model control (MC), positive control (PC), mulberry leaf powder (MLP), mulberry leaf fiber (MLF), mulberry leaf polyphenols (MLPS), mulberry leaf fiber and polyphenols mixture (MLM), and normal control (NC), and fed daily for 6 consecutive weeks. The results demonstrated that the MLM group had the best efficiency on weight loss, indicating synergistic interactions between MLPS and MLF. The reduction of *Firmicutes* abundance, and the downstream *Clostridiales*, *Lachnespiraceae*, was a key pathway for the antiobesity effects. The increased abundances of *Lactobacillus vaginalis* and *Lactobacillus gasseri* might result in lipid metabolism disorder. The test groups regulated the amino acid and oligopeptides metabolic disorder tents to normal levels compared with the MC and NC groups.

## 1. Introduction

Obesity, caused by a combination of genetic and environmental factors, is associated with nutritional metabolic disorders [1]. When energy intake in the body is greater than energy consumption, the imbalance causes fat to accumulate. Obesity affects body appearance and induces a variety of chronic diseases [2]. The rapid growth of obesity worldwide since the 1970s has been a major public health challenge [3]. Obesity is closely related to the intestinal bacterial community. Conventionalizing adult germ-free mice with normal microbiota harvested from the cecum of conventionally raised mice increased body fat content by 60% in 10–14 days [4].

Recent studies reported that some functional food components have weight loss effects and regulate body fat levels. These effects were associated with interactions between the intestinal community and nutrients [5]. The functional components in food are very complex and most are absorbed into the bloodstream after being ingested; however, some functional substances such as dietary fiber and polyphenols (prebiotics), enter the colon, interact with the colonic microbial community, and then exert their health effects [6]. Dietary fibers and polyphenols are found in almost all plant foods [7]. Some of these foods regulate the intestinal community, which causes weight loss and regulates lipid metabolism [8].

The mulberry leaf is an edible plant traditionally used to raise silkworms, which have been used as a medicine. The mulberry leaf has become increasingly popular as a healthy vegetable, especially in China, South Korea, Japan, Turkey, and other countries [9]. The hypoglycemic, hypotensive, regulation of vascular dysfunction, and weight loss effects of mulberry leaf were described in the pharmacopoeia. Furthermore, these functional activities have been verified by modern science with in vivo/in vitro experiments [9,10]. The mulberry leaf is also widely used in folk functional diets. The Compendium of Materia Medica records the effect of reducing body weight and fat after the long-term intake of the mulberry leaf. Mulberry leaf polyphenol extract (70% ethanol) did not affect body weight after 12 weeks of treatment, but had a regulatory effect on hepatic lipid metabolism, fibrosis, and antioxidant defense systems in high-fat diet-fed mice. Mulberry leaf water extract significantly decreased epididymal fat pad weight and ameliorated insulin sensitivity in the high-sucrose diet induced in over weight rats [11]. However, few studies have focused on the material basis and mechanism of the weight loss effects after ingesting mulberry leaf. A comparative study on the efficacy and interaction between mulberry leaf fiber and polyphenols has not been reported. In addition, the concomitant effect of mulberry leaf components on the intestinal microbiome and associated metabolites are unclear.

Therefore, in this study we established a nutritional obesity model, comparing the antiobesity effects of mulberry leaf powder, dietary fiber, polyphenols, and a fiber/polyphenols mixture. Combining intestinal community modulation and metabolite analysis, we investigated the antiobesity effects and mechanisms of mulberry leaf components, detecting the interaction between mulberry leaf dietary fiber and polyphenol.

## 2. Materials and Methods

### 2.1. Materials

Leaves of the mulberry tree (*Morus atropurpurea Roxb*, Da 10), collected from Baiyun National Mulberry Planting Resources Garden, Guandong Province, China in October, 2017, were identified and authenticated by the Guangdong Academy of Agricultural Sciences. SPF SD (Sprague-Dawley) male rats, weight 150 ± 20 g, were bought from Southern Medical University, Laboratory Animal Center, license number: SCXK (Guangdong) 2013-0034. Maintain feed (D12450-B, protein: 19.2%, carbohydrate: 57.3%, fat: 4.3%, energy: 3.85kcal/g), and high calorie feed (D12451, protein: 24%, carbohydrate: 41%, fat: 24%, energy: 4.73kcal/g) purchased from the Medical Experimental Animal Center of Guangdong Province, license number: SCXK (Guangdong) 2013-0002.

### 2.2. Preparation of Mulberry Leaf Polyphenol and Fiber Extracts

Mulberry leaf Polyphenols (MLPS) extract: mulberry leaves were dried, graded, and powdered into 60 meshes. Then, 100 g of leaf powder was extracted in 70% ethanol/water solvent with a concentration of 0.03 g/mL with ultrasonication (400 W). After 1 h of extraction, the mixture was centrifuged, and the supernatant was collected and concentrated with a rotary evaporation instrument. The MLPS extraction was harvested after freeze drying and the yield of MLPS extract was about 15 g with a polyphenols content of 20% in the extract as detected with Folin-reagent method. The high-performance liquid chromatography (HPLC) was carried out with our previous method [12].

Mulberry leaf fiber (MLF) extract: the above residue was added into distilled water to a concentration of 0.08 g/mL, a 1‰ volume concentration of thermal stable α-amylase was added and then placed in a constant temperature shaking water bath (95–100 °C) for 35 min. It was cooled to 60 °C, and a 2‰ volume concentration of protease solution was added with sustained oscillation for 30 min. Then, the pH value was adjusted to 4.5 ± 0.2 with hydroxide solution or hydrochloric acid solution, and a 2‰ amyloglucosidase solution was added and shaking was continued for another 30 min. The mixture was concentrated by rotary evaporation under reduced pressure, and 95% ethanol was added into the concentrated solution to make a final ethanol concentration of 75%. After centrifuging, the residue was dried to obtain MLF extract (about 60 g). Based on the extraction procedure, the MLF extract referred to the sum of water-soluble dietary fiber and insoluble dietary fiber [13]. The content of water-soluble dietary fiber is 5% and that of insoluble dietary fiber was 95%.

### 2.3. SD (Sprague-Dawley) Rats and Diet

The animal experiment was complied with the Animal Research Reporting in Vivo Experiments guidelines and carried out in accordance with the U.K. Animal and associated guideline. The experiment was conducted in the barrier animal room of the Guangdong Academy of Agricultural Sciences, Sericultural & Agri-Food Research Institute, with a license number of SYXK (Guangdong) 2015-0149. The ethic committee of the institute had approved the research in August 2018. The establishment of a high calorie induced obesity model: rats were fed with maintenance feed under a barrier system for 5–7 days (free intake of water, 20 °C, 12 h:12 h light-dark cycle). At the end of the adaptation period, rats were randomly divided into two groups according to body weight. Ten were given maintenance feed as the blank control group, and 120 rats were given the high calorie diet. Food intake and body weight were recorded once every week. Two weeks later, obesity sensitive rats were selected (diet-induced obesity resistant rats, rats with a low weight gain were eliminated) and continued to be fed with the high calorie diet for 6 weeks until the body weight increased to about 20% more than the blank control group, which was given maintenance feed.

The normal control group (NC) was continually fed with maintenance feed. Obese rats were randomly divided into 6 groups according to body weight, with 10 rats in each group: the obesity model control group (MC), positive control group (PC, gavage Orlistan tablet 0.0324 g/kg BW, commercial antiobesity medicine), mulberry leaf powder group (MLP, gavage 0.8 g/kg body weight (BW)), mulberry leaf fiber group (MLF, gavage 0.48 g/kg BW), mulberry leaf polyphenols group (MLPS, gavage 0.12 g/kg BW), and mulberry leaf fiber/polyphenols mixture group (MLM, gavage 0.6 g/kg BW, fiber: polyphenols 4:1). The doses of MLPS, MLF, and MLM were selected as equal to the content in MLP. Different experiment groups were gavaged once every day. In addition to gavaging, rats in the 6 groups had continual free access to a high calorie diet. Food intake and body weight were recorded for every week. Six weeks later, the feces and urine of each group were collected after gavage for 18 h and stored at −80 °C. Then, the rats were injected with 1% sodium pentobarbital (0.5 mL/100 g BW) to induce anesthesia and the body length was measured.

### 2.4. Tissue Harvest

Rats were dissected, and the blood and cecal contents were collected quickly. Fat and liver from rats were observed. Livers were separated and weighed. Body fat and the adipose tissue around the testis and prerenal area were collected and weighed, and the fat ratio was calculated with the formulas below [14]:

Fat index = fat weight/100 g BW

$$\mathrm{Lee}’s\;\mathrm{index}=\sqrt[{3}]{\mathrm{body}\;\mathrm{weight}\;\left(g\right)\times 10^{3}/\mathrm{body}\;\mathrm{length}\left(\mathrm{cm}\right)}$$

Liver index = liver weight/BW × 100%

All tissues and cecal contents were stored at −80 °C. Blood was centrifuged at 10,000 g at 4 °C for 15 min, and the supernatant was used to determine the blood index. A small piece of liver and adipose tissue was fixed with 4% paraformaldehyde, sliced, stained with hematoxylin-eosin (HE), and observed by a 400× microscope.

Plasma assays. Serum total cholesterol (TC), total triglycerides (TG), low-density lipoprotein cholesterol (LDL-C), high-density lipoprotein (HDL-C), and malondialdehyde (MDA) were detected with assay kits (Nanjing Jiancheng Institute of Biological Engineering, Nanjing, Jiangsu, China). All assays were performed according to the manufacturers’ instructions.

### 2.5. Cecal Microbiota

Cecal contents from the same group were sequenced and mixed. Microbial genomic DNA from cecal contents was isolated via a DNA isolation kit (CW0552S) purchased from Kangwei Shiji Biological Technology Co., Ltd. (Beijing, China), following the manufacturer’s protocol. DNA abundance was quantified using a Nanodrop5000 Ultramicro spectrophotometer (Beijing Baitaike Biological Technology Co., Ltd. Beijing, China). The V4 region of the 16S rRNA gene was amplified and sequenced using Illumina MiSeq (Illumina, San Diego, CA, USA). The primers employed were 27F 5′-ACTCCTACGGGAGGCAGCAG-3′ (forward primer) and 1492R 5′-GGACTACHVGGGTWTCTAAT-3′ (reverse primer). Based on the preliminary electrophoresis quantitative results, PCR products were quantified using Qubit fluorescence. Paired-end sequencing was performed on the Illumina platform (MiSeq) and low-quality reads (Q20, 90%) were filtered (Connecting Overlapped Pair-End, V1.2.3.3, San Diego, CA, USA).

### 2.6. Metabolomics

Urine and feces samples were extracted and prepared according to a previous protocol [15]. An ultra-performance liquid chromatography triple Time of Flight high resolution mass spectrometry (UPLC Triple TOF MS/MS) system from AB Sciex, Ltd. (Redwood, CA, USA), equipped with a liquid chromatography (LC) module (model AB ultraLC 110) coupled to a mass spectrometry (MS) module (model AB5600+) was used as a LC-MS platform. An Agilent 6890N gas chromatography (GC) equipped with Agilent 5975B mass spectrometry was used as the GC-MS platform. They were combined as an untargeted metabolomic approach and used to profile the urinary/fecal metabolites from SD rats.

### 2.7. Statistical Analysis

Operational Taxonomic Unit (OTU) analysis of microbiota species classification was performed with Ultra-fast sequence analysis (USEARCH) (V8.0.1623, San Diego, CA, USA) software. Then, the OTUs were further analyzed for abundance, diversity index, and the species community structure was evaluated at each classification level (Phylum, Class, Order, Family, Genus, Species). Then, QIIME (V1.8.0, San Diego, CA, USA) was used to determine the alpha diversity, chao1and beta diversity. The UPLC triple TOF MS/MS data were acquired and preprocessed using PeakView Software (Version 2.2, Redwood, CA, USA). The multivariate analyses of the metabolomics data were processed with partial least-squares-discriminate analysis (PSL-DA). Relative content ratios were normalized by the NC group or by the MC group when the NC group was not detected with the mean peak area. Significant differences (*p* < 0.05) were determined by Turkey’s test and an analysis of variance (ANOVA) using Statistical Product and Service Solutions (SPSS) software 9.0 for Windows (SPSS Inc., Chicago, IL, USA).

## 3. Results

### 3.1. The Determination of the Main Polyphenols in MLPS Extract

**Figure 1:**
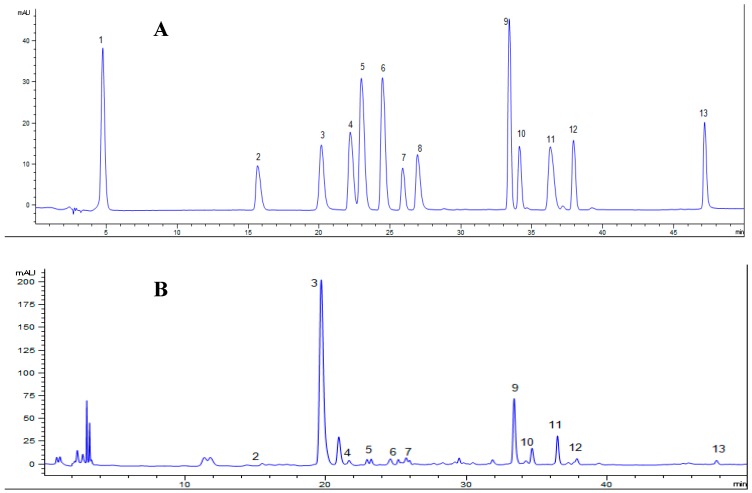
The high-performance liquid chromatography (HPLC) graph of mulberry leaf (Morus multicaulis Roxb) phenolic compounds (**A**) standards, (**B**) mulberry leaf Polyphenols (MLPS). Note: 1. gallic acid, 2. gentisic acid, 3. Chlorogenic acid, 4. vanillic acid, 5. caffeic acid, 6. syringic acid, 7. epicatechin, 8. Ferulic acid 9. rutin, 10. hyperoside, 11. benzoic acid, 12. Astragaloside, 13. Quercetin.

The quantitative and qualitative determination of polyphenols in MLPS extract was presented in Figure 1. The main phenolic compounds in mulberry leaves were chlorogenic acid, rutin, benzoic acid, and hyperoside with a content of 9.62, 4.04, 2.78, and 0.91 mg/g dried leaves.

### 3.2. Establisment of the Diet Induced Obesity (DIO) Model

Table 1 shows the body weight changes of the maintenance diet control group and high-energy diet model group. After 6 weeks of feeding, the average body weight of the high energy diet group was 20% higher than in the maintenance diet control group. Thus, the diet induced obesity (DIO) mouse model was established successfully.

**Figure 2:**
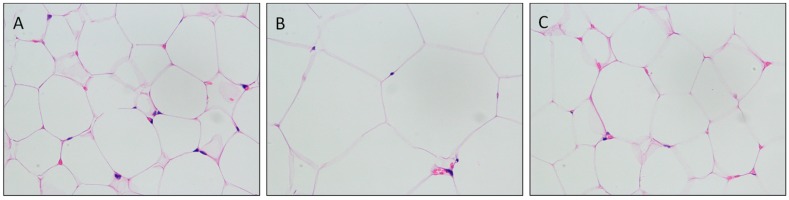
Hematoxylin-eosin (HE) staining slice of adipose tissue (400×). (**A)**, normal control (NC); (**B)**, model control (MC); (**C)**, positive control (PC).

After establishment of the dietary obese model, the rats were divided into 6 groups and administered different samples for another 6 weeks. A comparison of adipocyte HE staining in the NC, MC, and PC groups is shown in Figure 2. Adipocytes in the obese MC group were increased significantly, with a larger size compared with the NC and PC groups. In the same visual field, there were fewer adipocytes in the MC group compared with the other groups.

### 3.3. Mulberry Leaf Component Protected Rats from High Calorie Diet Induced Obesity

**Figure 3:**
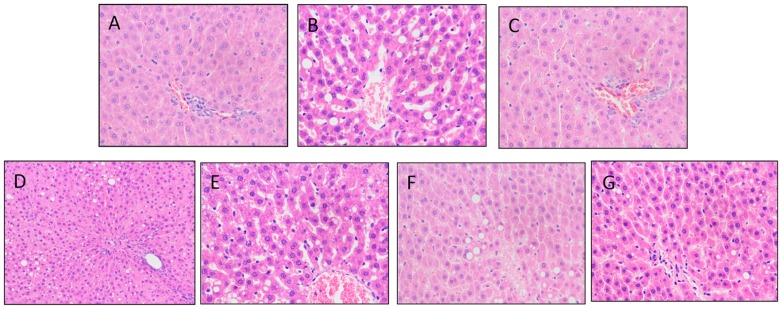
HE staining slice of liver tissues (400×). (**A**), normal control (NC); (**B**), model control (MC); (**C**), positive control (PC); (**D**), Mulberry leaf powder (MLP); (**E**), Mulberry leaf fiber (MLF); (**F**), Mulberry leaf polyphenols group (MLPS); (**G**), Mulberry leaf fiber/polyphenols mixture (MLM).

As shown in Figure 3, the hepatocyte fat of the MC group was degenerated, and a vacuole was clearly observed after fat dissolution. Under the administration of different samples by gavage, bubbles produced by fat lysis significantly decreased, and the hepatocyte gap was small and compact in the PC, MLF, and MLM groups compared with the MC group, which indicated that the amount of fatty liver was reduced in these groups. The vacuole in MLPS group could still be observed, indicating that the effect of MLPS was limited.

**Figure 4:**
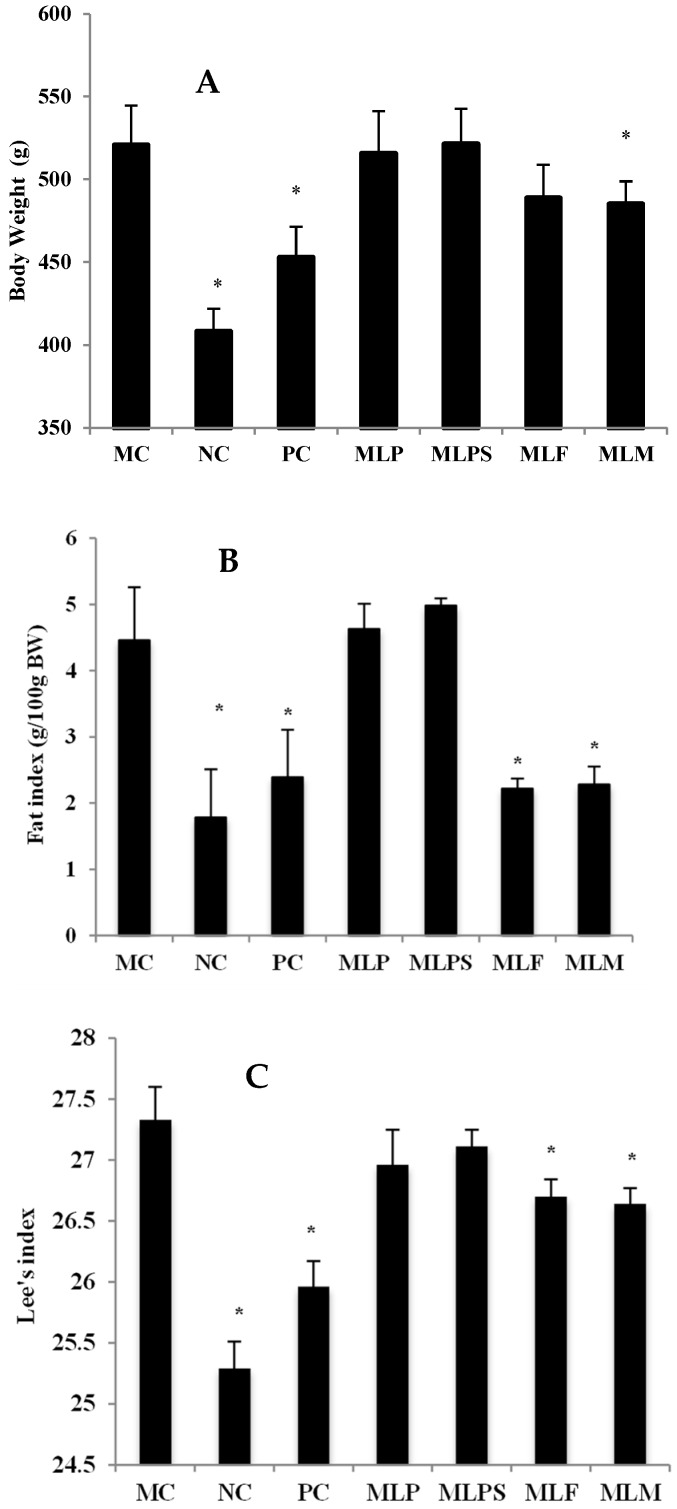
Body weight and index of different groups after gavage. (**A**), Body weight; (**B**), Fat index; (**C**), Lee’s index. *, *p* < 0.05 compared with the MC group.

There was no significant difference (*p* > 0.05) in the food intake, food utilization, and energy intake between each group (Table 2). Changes in the body weights of different groups after 6 weeks of sample administration were showed in Figure 4A. The NC and MC groups had a marked difference in body weight. Weight loss of the MLM group was the greatest among the test groups. After 6 weeks of MLM administration, the mean weight of this group was 485.0 g and a reduction of 6.9% body weight was observed compared with the MC group (520.7 g). The mean weight of the PC group was 452.9 g, indicating that intervention with the positive drug reduced the body weight of obese rats by 13.0%, and that MLM achieved half the efficacy of the positive drug. The MLF and MLP groups also reduced the body weight of obese rats to some extent after 6 weeks administration with a mean weight of 488.8 g and 515.7 g respectively, although the difference did not reach statistical significance. However, intervention with MLPS increased the weight of obese rats to 552.4 g and 521.3 g at weeks 5 and 6, respectively, indicating that MLPS did not have a weight loss effect.

A significant difference between the NC and MC groups was observed in the fat index and Lee’s index. Compared with the MC group, the MLF, MLM and PC groups showed a decrease in the fat index and Lee’s index, while the MLP and MLPS test groups did not show a significant effect (Figure 4B,C).

### 3.4. Mulberry Leaf Components Modulated Lipid Metabolism Disorder in DIO Serum

**Figure 5:**
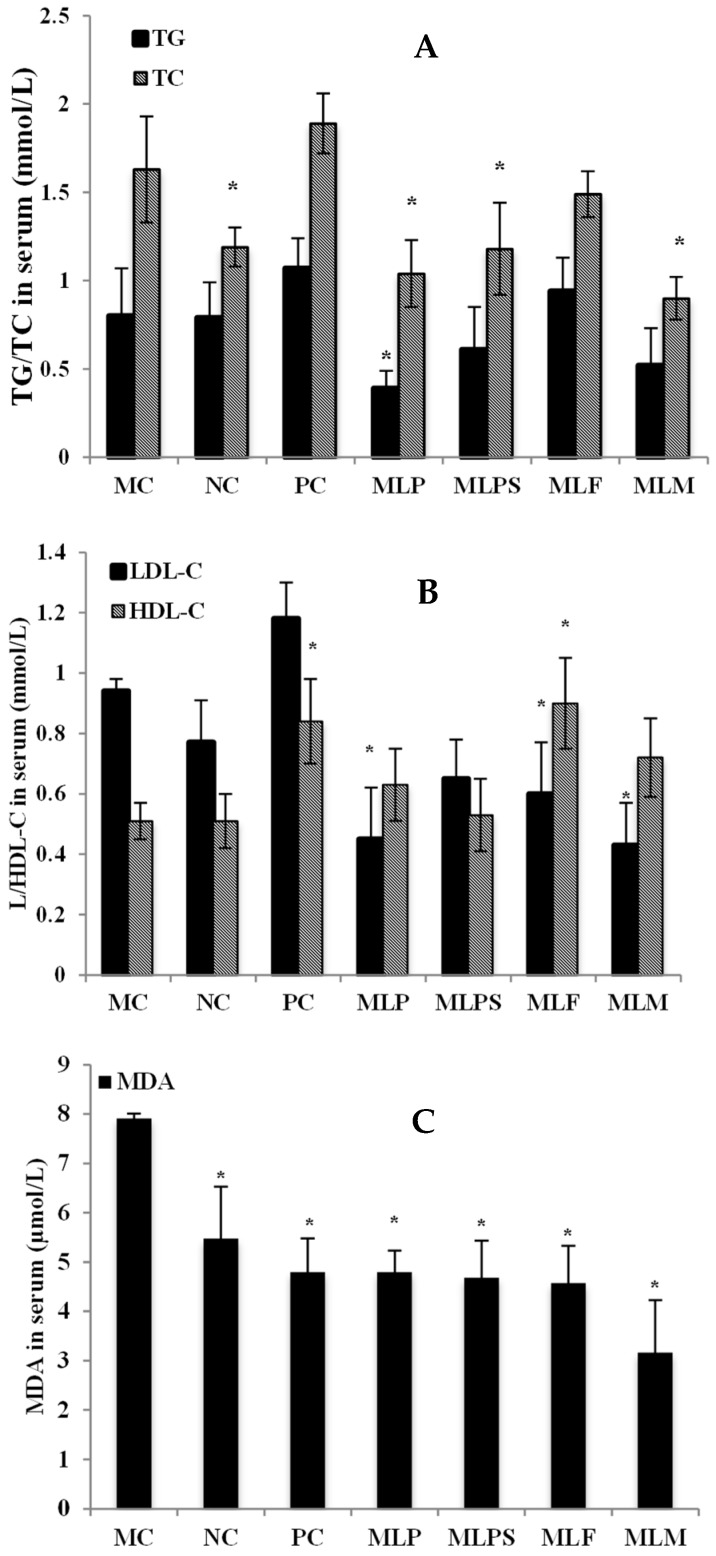
Serum index of different groups after gavage. (**A**), Serum total cholesterol (TG) and total triglycerides (TC); (**B**), low-density lipoprotein cholesterol (LDL-C) and high-density lipoprotein cholesterol (HDL-C); (**C**), malondialdehyde (MDA). *, *p* < 0.05 compared with the MC group.

No significant difference appeared in TG content between the NC and MC groups, while the TC content in the NC group was significantly lower than in the MC group. Compared with the MC group, the test groups showed no significant effect on the TG content, except the MLP group, which showed a decrease in the TG content of obese rats. In addition to the MLF group, MLP, MLPS and MLM significantly reduced the TC content in the serum of obese rats, while the TC content in the PC group was increased slightly without significance (Figure 5A).

Although the LDL-C and HDL-C levels in the MC group were not significantly different from those in the NC group, the content of LDL-C was decreased significantly in the MLP, MLF, and MLM groups, and the HDL-C content was increased significantly in the PC and MLF groups (Figure 5B) compared with MC. The MDA content in the serum of all experiment groups was significantly reduced (Figure 5C), showing that the mulberry leaves and their components had different degrees of anti-oxidation/free radical scavenging effects compared with the MC group. Among these groups, the antioxidant effect of the MLM was the strongest when the MDA content in the serum decreased to 3.16 µmol/L.

The mulberry leaf and its components had different degrees of effects on weight loss/lipid metabolism regulation. The significant effectiveness of different group is concluded in Table 3. MLF had a weight loss effect while MLPS showed none. As a combination of MLF and MLPS, MLM showed the best efficiency, indicating that the mulberry fiber and polyphenols had a synergistic interaction.

### 3.5. DIO Gut Environment is Altered by Mulberry Leaf Components Supplement

**Figure 6:**
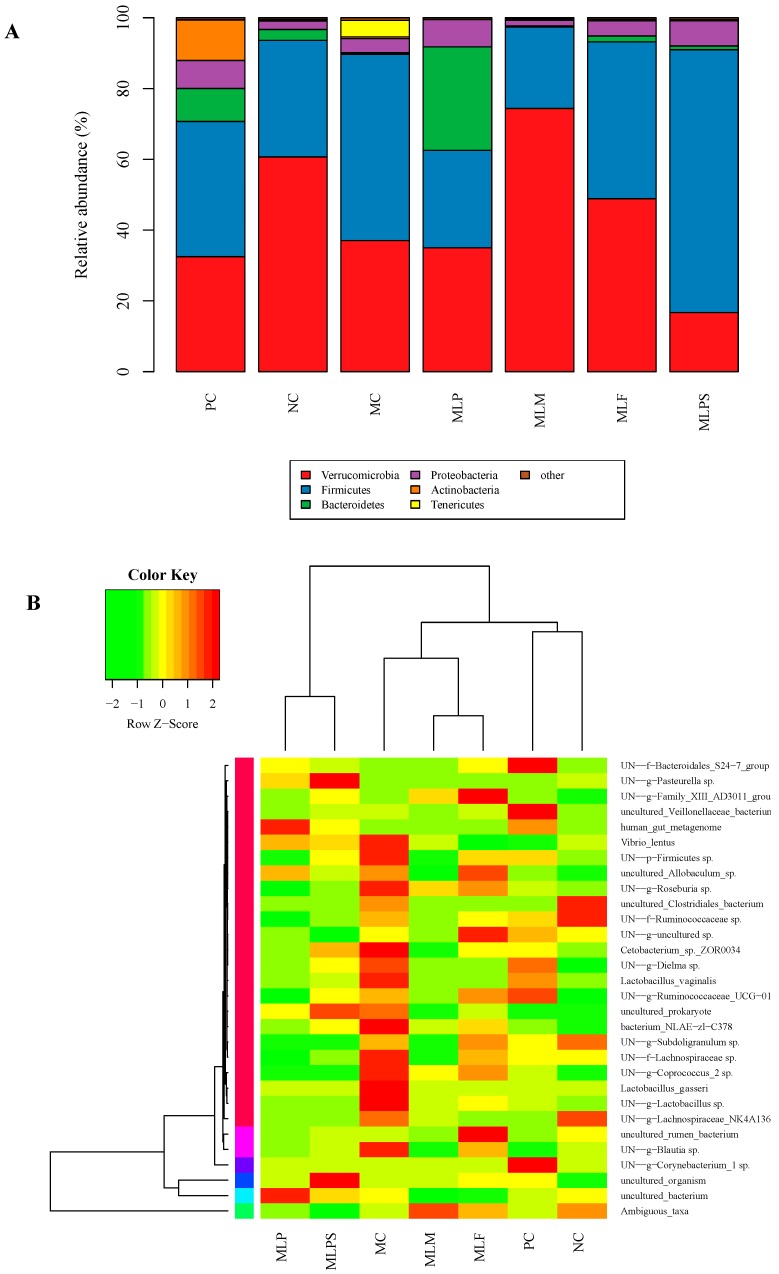

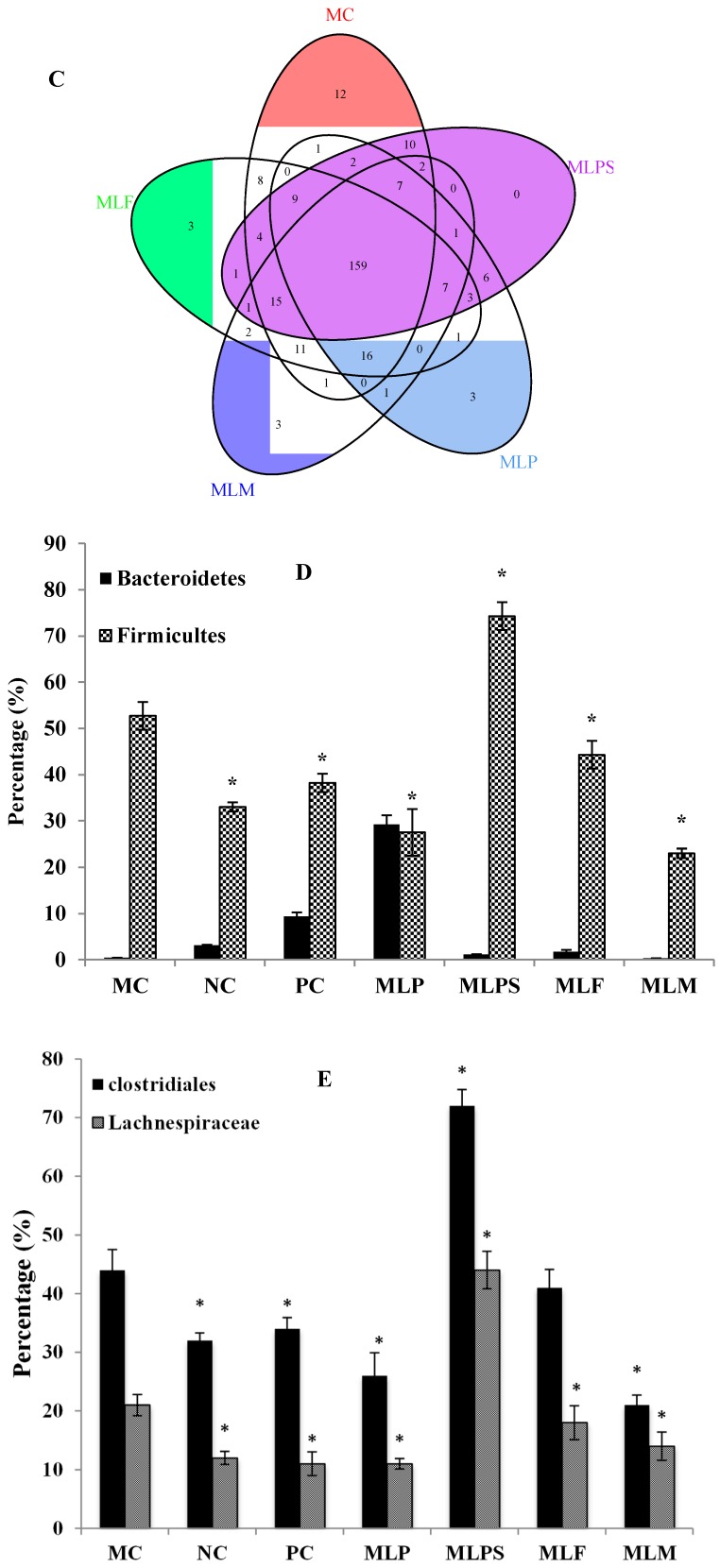

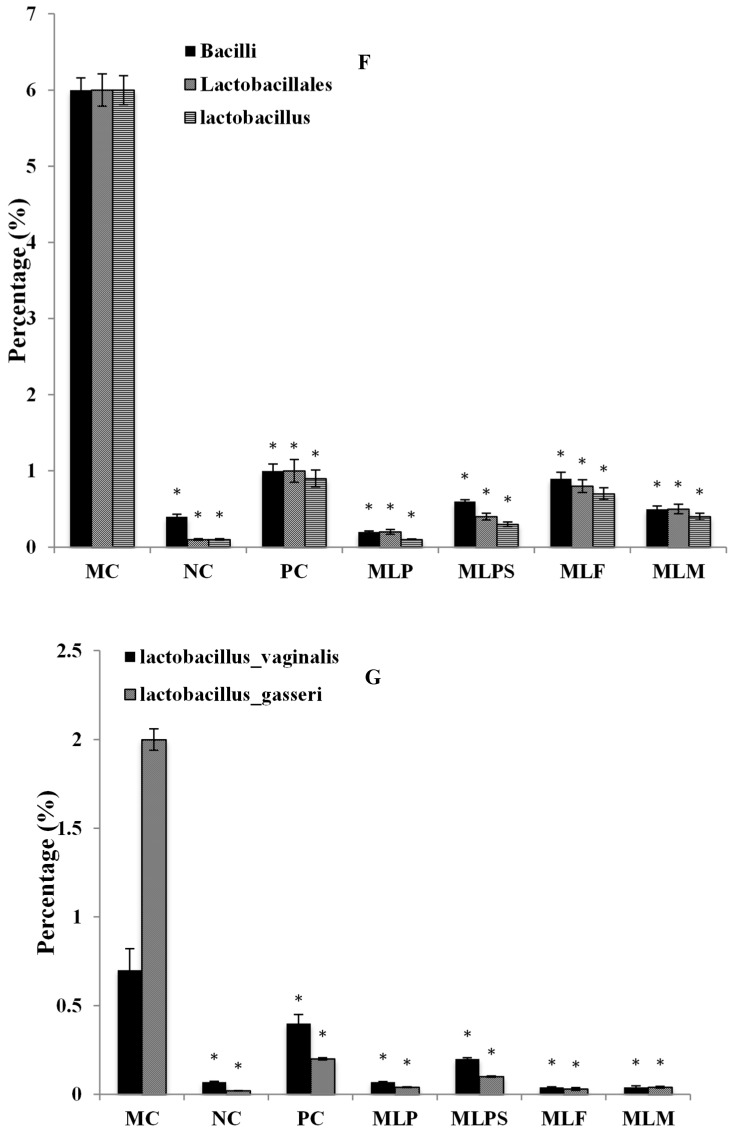
Mulberry leaf components change the gut microbiota composition among different groups after gavage. (**A**), Cecal microbiota at phylum level; (**B**), heap maps at species level/heat map information of the 30 most abundant species among different groups; (**C**), The Venn diagrams; (**D**), Percentage of *Firmicutes* and *Bacteroidetes* in different groups; (**E**), *Clostridiales* and *Lachnespiraceae* abundance among different groups. (**F**), *Bacillus*, *Lactobacillales* and *Lactobacillus* abundance among different groups; (**G**), *Lactobacillus vaginalis* and *Lactobacillus gasseri* abundance among different groups. *, *p* < 0.05 compared with the MC group.

The intestinal microbial community structure of different test groups was analyzed at the level of classes, orders, gates, families, genera, and species, and relative abundance bar charts (Figure 6A) and heat maps (Figure 6B) were obtained. The community structure of the gut microbials was altered by the high calorie diet, which further affected the lipid metabolism balance and the development of obesity. Venn diagrams were obtained by comparing the Operational Taxonomic Units (OTU) among 4 experiment intervention groups (MLP, MLPS, MLF, MLM) and the MC group, to determine the gut microorganism richness (Figure 6C). The intestinal microbial community of the 5 groups was different, and the MLPS group showed the smallest microbial abundance.

Compared with the NC group, *Bacteroidetes* in the MC group were reduced while *Firmicutes* increased significantly (Figure 6D). It is observed that the proportion of *Firmicutes/Bacteroidetes* in obese individuals (MC) is higher than in normal individuals (NC). Because of the low proportion of *Bacteroidetes* in the different groups (0.3% MC, 3% NC), we focused on differences in *Firmicutes* among the different groups. Compared with the MC group, other groups (MLP, MLF, MLM, PC but not MLPS) showed a reduction of *Firmicutes* abundance to the level of the NC group. Besides, the efficiency of MLM administration was the greatest among all groups. These results were consistent with the weight loss effect in each group.

Compared with the MC and MLPS groups, the content of *Firmicutes*, *Clostridiales*, and *Lachnespiraceae* were decreased in the gut microorganisms of the PC, MLP, MLF, and MLM groups to the normal levels observed in the NC group (Figure 6E). The *Firmicutes* content in the MC, NC, PC, MLP, MLPS, MLF, and MLM groups was 53, 33, 38, 28, 74, 44, and 23% respectively. The *Clostridiales* abundance was 44, 32, 34, 26, 72, 41, and 21% respectively, and the *Lachnespiraceae* content was 21, 12, 11, 11, 44, 18, and 14% respectively. The weight loss efficacy of MLPS was not obvious, and the body weight of obese rats was increased by MLPS to some extent. Other test groups might achieve weight loss (reduced body weight, fat index or Lee’s index) by significantly inhibiting the abundance of *Lachnespiraceae*, *Clostridiales*, and *Firmicutes* in the intestinal microbiota community.

As shown in Figure 6F,G, the abundances of *Bacillus*, *Lactobacillales*, *Lactobacillus*, and the species of *Lactobacillus vaginalis* and *Lactobacillus gasseri* were significantly reduced in the PC, MLP, MLPS, MLF, and MLM groups compared with the MC group. Including MLPS, all experiment groups showed a significant regulatory effect on lipid metabolism (TG, TC, HDL-C, or LDL-C) and a serum free radical scavenging effect, which may be related to the inhibitory effect on Bacillus (belonging to *Firmicutes*), *Lactobacillales*, *Lactobacillus*, *Lactobacillus vaginalis*, and *Lactobacillus gasseri* content in the gut microflora.

### 3.6. Urinary and Fecal Metabolites

In this study, the biological function and metabolics of rats were changed by a high calorie diet (Table 4 and Table 5). The MC group rats showed a marked difference (with large fold changes) in amino acid metabolism, purine-pyrimidene metabolism, oligopeptide metabolism, carbohydrate metabolism, and other important metabolic pathways compared with the NC group. The differences in urine and fecal metabolites suggested that the high energy diet caused unhealthy metabolic disorders. In obese rats treated with different components from mulberry leaves (MLP, MLF, MLPS, MLM), the relative content of the metabolites trended toward the level observed in the NC group, showing a certain degree of regulatory effects. Some metabolites related to lipid metabolism were screened. The related representative urinary metabolites were 2-indolecarboxylic acid, indoleacetic acid, shikimic acid, thyronine, homocysteine, glycine, 5-aminopentanoic acid, phenylalanyl-hydroxyproline, arabitol, arabinose, glycylprolyl-hydroxyproline, and phenylalanyl-hydroxyproline. The relevant representative fecal metabolites were nicotinic acid, 2-oxo-4-methylthiobutyric acid, isoleucine, methylguanine, xanthine and D-1-piperideine-2-carboxylic acid.

MLPS exhibited the poorest weight loss effect, while other test groups experienced different degrees of weight loss. The urine metabolites that had a greater correlation with weight loss were thyronine, glycine, arabinose, and phenylalanyl-hydroxyproline. The fecal metabolites with the same trend as this result were methylimidazole acetic acid threonic acid, propionate, and butyrate. Although significant weight loss was observed in the PC group, the metabolites expressed huge differences with the MC and NC groups, suggesting that metabolic disorders can be induced by positive drugs (PC). For example, the relative contents of urine indoleacetic acid and methylguanine were increased to 3.6 and 5.33, respectively, and the fecal nicotinic acid abundance decreased to 0.41. These results were consistent with the side effects of Orlistat as a commercial antiobesity drug.

## 4. Discussion

The present study provides evidence that the mulberry leaf and its components have different degrees of weight loss/lipid metabolism regulatory effects. The MLP reduced serum TG, TC, LDL-C, and MDA content in obese rats. MLPS only decreased serum TG and MDA content. MLF lowered the fat index, Lee’s index, and serum LDL-C and MDA, as well as increasing serum HDL-C. MLM had a significant beneficial effect on reducing rat body weight, fat index, Lee’s index, and serum TG, LDL-C, and MDA.

The mulberry leaf possesses anti-obesity and lipid metabolic modulation effects as per previous reports [9,11,16]. Most experiments have focused on the efficacy of mulberry water extract [11], ethanol extract [17], or particular compounds, such as 1-Deoxynojirimycin, resveratrol, and quercetin 3-(6-malonyglucoside) [18]. The mulberry leaf polyphenol extract (70% ethanol extract) decreased liver lipid peroxidation levels and adipocyte size without body weight loss, while the combined mulberry leaf and fruit extract significantly decreased body weight gain through modulation of obesity-induced inflammation and oxidative stress in high fat diet induced obesity [16] To the best of our knowledge, no previous study has investigated the combined effect of mulberry polyphenols and fiber on obesity, and how the mulberry leaf and its components modulate gut microbiota and metabonomics.

The effect of MLF on obesity was demonstrated for the first time in the present study. Many studies have demonstrated that various kinds of dietary fibers have antiobesity and hypolipidemic effects [19,20,21]. In a study of the weight reducing effect of apples, it was demonstrated that apple-derived soluble fiber suppressed weight gain and fat accumulation in diet induced obese rats, and modulated the gut microbiota, metabolic endotoxemia, and systemic inflammation [22]. A proprietary herb and fiber combination group significantly reduced the body weights and perirenal white adipose tissue compared with the high fat induced obesity group [19]. The results of the MLPS group were consistent with the results of Ann et al. [9]. After 12 weeks of treatment with MLPS, there was no obvious decrease in body weight, but obesity-induced hepatic lipogenesis, fibrosis, and oxidative stress were ameliorated in high fat diet-fed rats.

The molecular interactions between dietary fiber and polyphenols can affect their bioavailability and beneficial effects in the food matrix [23]. The main interaction forces involved are van der Waals forces, including hydrogen bonding, London interaction, and ionic interaction. In the experiment, polyphenols were separated from dietary fiber in the first step and then combined at a ratio of 1:4 in the MLM group. MLM exhibited the strongest effect among all mulberry leaf components. MLPS and MLF were released from each other and showed health benefits because of their improved bioaccessibility. Furthermore, both bioactive compounds may interact with other food components in the gut and form chemical complexes and colloidal structures via binding forces that improve their bioactivity [24]. The functional ingredients in MLP, including dietary fiber and polyphenols, were tightly compacted, which resulted in a low utilization rate and being discharged directly into the intestine. In addition, the protein, fat and other components hydrolyzed during the fiber extraction process had a negative effect on weight loss. Thus, the weight suppressing effect of MLP was not significant compared with MLM. Both MLP and MLM had a marked lipid metabolism modulating effect, which related to the inhibition of absorption by forming complexes with lipid and lipolytic enzymes [25].

*Lachnespiraceae*, belonging to *Firmicutes/Clostridiales*, was abundant higher in the intestine and was relatively rare elsewhere. Our results suggested that a high calorie diet induced dynamic changes in the *Lachnespiraceae* composition. Compared with the NC group, the MC group had higher proportions of *Lachnespiraceae*. The other experiment groups (PC, MLP, MLF, MLM, except MLPS) might resist developing obesity by regulating the content and composition of *Lachnespiraceae* in the gut bacteria. Our results were consistent with a study by Zeng et al. [26], who demonstrated that long-term high fat consumption increased body weight and inflammatory status as well as the abundance of *Lachnespiraceae* bacteria in the hind gut of C56BL/6 rats. A candidate bacterium was isolated from the feces of hyperglycemic obese rats and identified as *Lachnespiraceae* (strain AJ110941). The colonization of *Lachnespiraceae* (strain AJ110941) to germ free ob/ob rats, induced significant adipose tissue weight, blood glucose levels, and plasma insulin levels, indicating that AJ110941 was related to the development of obesity and diabetes [27]. *Lachnospiraceas* have been linked to obesity, most likely because of their ability to inhibit short chain fatty acid producing strains [28], and they were reported to be involved in the development of type II diabetes, metabolic disorders, and colon cancer [27,29]. Polyphenols simulate the favorable effects of fiber and prebiotics on the gut bacterial content [30]. However, the molecular mechanisms underlying their positive effect on obesity needs further study.

Our study found that in the high energy diet induced MC group, the abundance of the *Bacillus* (belonging to *Firmicutes*), *Lactobacillales*, *Lactobacillus*, and species of *Lactobacillus vaginalis* and *Lactobacillus gasseri* were increased significantly compared with the NC group. After adding the mulberry leaf components to the diet, the abundance of these species in the intestines was similar to the level found in the NC group. Unlike other Lactobacillus probiotics, our study demonstrated for the first time that the increase in the abundance of two *Lactobacillus* species (*vaginalis* and *gasseri*), was closely related to lipid metabolism disorder in obese rats. Few previous studies have investigated the effect of gut bacterial intervention on lipid metabolism. Some *Lactobacillus* species were associated with lipid metabolism regulation and acted as probiotics, while other *Lactobacilli* were associated with the lipid metabolism disorder [31]. Milk fermented by *Lactobacillus* strains exhibited a hypocholesterolemic effect in hamsters by lowering serum and liver total cholesterol levels [32]. Lactobacillus plantarum (LS/07 and Biocenol LP96) improved the lipid profile of high fat diet fed rats without a significant change in body weight [33]. The present results indicate that, although the majority of *Lactobacillus* were probiotics, a high content of *Lactobacillales/Lactobacillus* in the gut microbes did not have better benefit in terms of lipid metabolism. Thus, the content of *Lactobacillales/Lactobacillus* should be maintained at a normal level to maintain health. In the MC group, high calorie feeding caused obesity, and a significant increase in gut *Lactobacillus* content altered the intestinal microbial balance, inducing lipid metabolic disorder. The addition of mulberry leaf components and positive drugs adjusted the content of *Lactobacillus* to normal levels.

Furthermore, fecal and urine metabolites were altered by feeding with a high calorie diet and the disorder was positively regulated among different groups. Gut bacteria influenced the host metabolism through multiple pathways, which interacted with the transformation, absorption, and metabolism of exogenous substances. Substances that differed only in the feces were directly from the intestinal microbial community and were mostly excluded from the body; substances that differed both in the feces and urine entered the body circulation after intestinal microbial metabolism, which affected metabolism in systemic circulation; the substances that differed only in the urine did not differ because of the direct metabolites of the intestinal microbial community, but rather because of the absorption metabolism at cecal level and the upper gastrointestinal tract, or synergistic/compensatory changes in the systemic circulation of changes in other metabolites.

Indoles (2-indolecarboxylic acid, indoleacetic acid) were produced by the fermentation of aromatic tryptophan. In particular, tryptophan invertase, secreted by *E. coli*, converts tryptophan into indoles, which is absorbed into the blood through the intestine and excreted from the urine [34]. Indoles, traditionally recognized for its toxicity on gut bacteria [35], showed beneficial effects from the recent research [36]. The histidine content in the MC group was significantly higher than in the normal group, indicating that the high calorie diet inhibited the conversion of histidine by inhibiting the growth of the relevant microbial community that secreted histidine invertase. Urocanic acid is a metabolite that is generated during histidine conversion, both in urine and feces. In the PC group, the amount of urocanic acid was greatly increased compared with the MC and NC groups, which was the result of the promotion of the relevant gut bacteria growth [37]. The production of short chain fatty acid, propionate, and butyrate was inhibited in the MC group but was modulated in the test sample groups. The content of the butyrate in the MLM group was the highest, which was consistent with its antiobesity effect. Butyrate increased the energy expenditure in dietary-obese mice, which resulted in reduced body weight [38]. The oligopeptides in the MC group had higher degradation compared with those in the NC group. Oligopeptides have more active functions compared with amino acids, as in previous reports [39]. Compared with the NC group, high calorie diet feeding significantly reduced the oligopeptide content in the MC group, including urine aspartyl-glutamic acid, aspartyl-leucine, phenylalanyl-hydroxyproline, and glycylprolyl-hydroxyproline. In the test sample groups, health benefits could be exerted by regulating an oligopeptide metabolism disorder. However, the molecular mechanisms involved in the metabolites related to obesity and lipid metabolism still need further research.

## 5. Conclusions

The present study compared the treatment of MLP, MLPS, MLF, and MLM on obesity and lipid metabolism related indexes in DIO rats together with the MC, PC, and NC groups. The synergistic interaction between mulberry dietary fiber and polyphenols (MLM) in antiobesity was reported for the first time. The addition of mulberry leaf components had a marked influence on the structure of the intestinal community. Among them, the content of *Firmicutes* in the MC group was increased significantly. Except for the MLPS group, other test groups regulated the *Firmicutes* content to a normal level. Our study demonstrated that different components of mulberry leaves might achieve weight loss by reducing the amount of *Lachnespiraceae* (belonging to *Firmicutes/Clostridiales*). At the same time, the reduction of Bacillus, *Lactobacillales*, *Lactobacillus*, *Lactobacillus_vaginalis* and *Lactobacillus_gasseri* species was closely related to the improvement of lipid metabolism profiles. In addition, the high energy diet induced feces and urine metabolic disorders in the MC group with significant difference. The amino acid and oligopeptide metabolites were regulated to the NC level under the regulation of mulberry leaf components in the test groups.

## Figures and Tables

**Table 1:** Body weight changes during model establishment.

Weeks	Maintain Diet Group (g)	High-Energy Diet Group (g)
1	240.2 ± 20.7	244.6 ± 13.7
2	276.4 ± 29.8	300.6 ± 8.8
3	312.1 ± 17.3	346.7 ± 10.4
4	348.1 ± 18.8	381.4 ± 12.1
5	365.4 ± 12.9	420.3 ± 15.9
6	370.6 ± 13.1	442.8 ± 11.7

**Table 2:** Food intake and utilization of each group during gavage.

	MC	PC	MLP	MLPS	MLF	MLM
Intake(g)	17.80 ± 0.43	17.83 ± 1.44	17.37 ± 0.71	17.21 ± 1.17	16.12 ± 1.12	16.69 ± 1.03
Utilization%	11.05 ± 1.23	2.16 ± 0.76	8.87 ± 0.77	11.43 ± 1.54	7.04 ± 0.68	9.03 ± 0.97

MC: obesity model control; PC: positive control; MLP: mulberry leaf powder; MLF: mulberry leaf fiber; MLPS: mulberry leaf polyphenols; MLM: mulberry leaf fiber and polyphenols mixture. Note: food utilization refers to the ratio of body weight gain and food intake (*p* > 0.05, no significant difference).

**Table 3:** The significant effectiveness of different group.

	MLP	MLPS	MLF	MLM
Body weight	×	×	×	↓
Fat index	×	×	↓	↓
Lee’s index	×	×	↓	↓
TG	↓	×	×	×
TC	↓	↓	×	↓
HDL-C	×	×	↑	×
LDL-C	↓	×	↓	↓
MDA	↓	↓	↓	↓

Note: “×”indicates no significant difference compared with MC group. “↓” means a significant decrease compared with MC group and “↑” means significant increased compared with MC group.

**Table 4:** Representative and main differentiated urinary metabolites derived from mammalian-gut microbial co-metabolism among different groups analyzed by UPLC-Triple-TOF-MS and GC-MS.

No.	Metabolites	The Relative Content						
		MC	NC	PC	MLP	MLPS	MLF	MLM
Tryptophan								
1	2-Indolecarboxylic acid	4.53 ^#^	1	3.14 *	0.29 *	0.19 *	0.82 *	0.52 *
2	Indoleacetic acid	0.40 ^#^	1	3.60 *	0.63 *	0.78 *	0.57 *	0.86 *
Tyrosine and phenylalanine								
3	Tyrosine	1.55 ^#^	1	0.75 *	0.93 *	1.09 *	1.60	1.31
4	Shikimic acid	2.67 ^#^	1	1.95 *	1.36 *	1.20 *	0.74 *	0.62 *
5	Thyronine	0.20 ^#^	1	1.55 *	0.47 *	0.35 *	0.47 *	0.83 *
Histidine								
6	Urocanic acid	1.07	1	2.33 *	0.93	1.64 *	1.47 *	0.74 *
Cysteine/Methionine								
7	Pyroglutamic acid	0.40 ^#^	1	4.33 *	0.39	0.21 *	0.84 *	0.45 *
8	Homocysteine	0.54 ^#^	1	3.74 *	0.83*	1.08 *	0.58	0.62 *
9	Methionine	1.53 ^#^	1	4.31 *	-	1.04 *	1.29 *	0.95 *
Valine/leucine/isoleucine/lysine								
10	Glycine	0.39 ^#^	1	1.32 *	0.52 *	0.49 *	0.69 *	0.62 *
Arginine/Proline								
11	5-Aminopentanoic acid	0.52 ^#^	1	0.30 *	0.58 *	0.74 *	0.35	1.10 *
Purine/pyrimidine								
12	Methylguanine	1.37 ^#^	1	5.33 *	1.31	0.53 *	1.14 *	1.97 *
13	Deoxycytidine	1.08	1	0.29 *	0.91	2.77 *	2.15 *	0.63 *
Oligopeptides								
14	Aspartyl-glutamic acid	0.37 ^#^	1	0.36	0.49 *	0.40	0.80 *	0.46
15	Aspartyl-leucine	-	1	1.13	0.17 *	0.14 *	0.43 *	0.23 *
16	Leucyl-proline	0.88 ^#^	1	0.11 *	1.02	1.60 *	2.53 *	1.02
17	Phenylalanyl-hydroxyproline	0.02 ^#^	1	0.48 *	0.21 *	0.17 *	1.10 *	0.45 *
18	Prolyl-glycine	1.39 ^#^	1	2.01	-	1.47	-	-
Carbohydrate								
19	Arabitol	0.46 ^#^	1	1.29 *	0.78 *	1.21 *	0.85 *	0.75 *
20	Arabinose	0.40 ^#^	1	1.64 *	1.06 *	0.28 *	1.11 *	1.25 *
Others								
21	Glycylprolyl-hydroxyproline	0.41 ^#^	1	2.57 *	0.63 *	1.32 *	1.41 *	1.22 *
22	Ophthalmic acid	-	1	3.80 *	0.39 *	0.57 *	0.61 *	0.50 *

Metabolites selected by *p* < 0.05 in one-way ANOVA. (-) indicates not detected. Relative content ratios were normalized by the NC group or by the MC group (when NC group was not detected; -) with the mean peak area. The symbol “*” indicates statistically significant the test groups compared with the MC group. The symbol “^#^” indicates a statistically significant MC compared with the NC group.

**Table 5:** Representative and main differentiated fecal metabolites derived from mammalian-gut microbial co-metabolism among different groups analyzed by UPLC-Triple-TOF-MS and GC-MS.

No.	Metabolites	The Relative Content						
		MC	NC	PC	MLP	MLPS	MLF	MLM
Tryptophan								
1	Tryptophan	1.78 ^#^	1	0.76 *	2.59 *	0.76 *	3.47 *	1.10 *
2	2-Indolecarboxylic acid	1.10	1	0.57 *	0.39 *	0.88 *	1.16	0.88 *
3	Nicotinic acid	32.70 ^#^	1	0.41 *	6.59 *	1.56 *	20.89 *	14.10 *
Tyrosine and phenylalanine								
4	Tyrosine	2.09 ^#^	1	3.08 *	2.89 *	0.33 *	2.93 *	1.33 *
5	2-Phenylacetamide	1.92 ^#^	1	5.30 *	0.96	0.36 *	2.75 *	1.13
6	Phenyl-acetaldehyde	2.24 ^#^	1	16.50 *	-	2.69 *	2.47	-
7	Phenylacetic acid	4.52 ^#^	1	26.82 *	-	4.24	5.19 *	2.71 *
Histidine								
8	Urocanic acid	0.41 ^#^	1	0.11 *	0.53 *	0.31 *	1.85 *	-
9	Histidine	6.01 ^#^	1	4.12 *	1.04 *	-	-	3.59 *
10	Methylimidazoleacetic acid	4.71 ^#^	1	2.75 *	0.97 *	1.74 *	1.43 *	0.83 *
Cysteine/ Methionine								
11	2-Oxo-4-methylthiobutyric acid	0.39 ^#^	1	2.26 *	1.40 *	2.25 *	1.79 *	0.77 *
Valine/leucine/isoleucine/lysine								
12	Isoleucine	2.23 ^#^	1	1.29 *	1.84 *	0.98 *	1.94 *	1.36 *
Arginine/Proline								
13	Creatinine	0.06 ^#^	1	4.53 *	2.76 *	-	0.02	1.21 *
14	Glutamic acid	0.91	1	14.89 *	-	5.38 *	5.19 *	0.65 *
Purine/pyrimidine								
15	Methylguanine	0.58 ^#^	1	1.38 *	2.23 *	0.68 *	2.09 *	1.13 *
16	Xanthine	0.28 ^#^	1	0.94 *	0.33 *	0.67 *	0.59 *	0.51 *
17	Adenine	0.03 ^#^	1	1.13 *	0.04	-	-	0.46 *
Carbohydrate								
18	Threonic acid	3.85 ^#^	1	3.91	1.59 *	7.85 *	6.89 *	1.80 *
SCFAs								
19	Acetate	1.20	1	1.5 *	0.78 *	1.23 *	0.87 *	0.96
20	propionate	0.50 ^#^	1	0.61 *	0.71 *	0.55	0.63 *	0.67 *
21	butyrate	0.37 ^#^	1	0.62 *	0.48 *	0.26	0.72 *	0.81 *
Others								
22	Argininic acid	1.95 ^#^	1	0.96 *	-	9.71 *	16.87 *	0.85 *
23	D-1-Piperideine-2-carboxylic acid	5.04 ^#^	1	2.51 *	1.41 *	1.46 *	2.23 *	-
24	Ophthalmic acid	1	-	0.39 *	0.57 *	1.31 *	1.99 *	-
25	Phenylalanyl-hydroxyproline	1.61 ^#^	1	9.56 *	6.69 *	0.60 *	2.31 *	2.23 *
26	Carnitine	-	1	5.36 *	0.67 *	0.74 *	1.00	0.69 *

Metabolites selected by *p* < 0.05 in one-way ANOVA. (-) indicates not detected. Relative content ratios were normalized by the NC group or by the MC group (when NC group was not detected; -) with the mean peak area. The symbol “*” indicates statistically significant test groups compared with the MC group. The symbol “^#^” indicates a statistically significant MC compared with the NC group.
